# Human-animal interaction: understanding the role of dog and cat interactions in emotional wellbeing

**DOI:** 10.3389/fpsyg.2026.1768288

**Published:** 2026-06-16

**Authors:** Sanne Peeters, Nele Jacobs, Karin Hediger, Jannes Eshuis, Mayke Janssens

**Affiliations:** 1 Department of Lifespan Psychology, Faculty of Psychology, Open University, Heerlen, Netherlands; 2 Department of Psychiatry and Psychology, School for Mental Health and Neuroscience, Maastricht University Medical Centre, Maastricht, Netherlands; 3 Department of Clinical Psychology, Faculty of Psychology, Open University, Heerlen, Netherlands; 4 Division of Clinical Psychology and Animal Assisted Interventions, Faculty of Psychology, University of Basel, Basel, Switzerland; 5 Department of Theory Methods and Statistics, Faculty of Psychology, Open University, Heerlen, Netherlands

**Keywords:** companion animals, emotional-wellbeing, experience sampling method (ESM), human-animal interaction, negative affect, positive affect, stress-buffering

## Abstract

Companion animals are often assumed to benefit human emotional wellbeing, yet empirical evidence for this effect and the proposed stress-buffering mechanism underlying this effect is heterogeneous. This study examined whether momentary interaction with a companion animal is associated with pet owners’ positive and negative affect in daily life, and whether these interactions buffer the affective impact of stress. We further tested whether these associations differ between interacting with dogs versus cats. Using ecological momentary assessment (EMA), 188 dog and cat owners reported their affect, stress, and interactions with their companion animals at random moments up to 10 times per day over five consecutive days. Multilevel regression analyses accounting for repeated measurements nested within individuals, including random intercepts and slopes, and controlling for age, gender, and social context, showed that pet interaction was associated with higher positive and lower negative affect, independent of species. No evidence for a stress-buffering effect was found, instead a species-specific pattern emerged for negative affect in response to event-related stress: interactions with cats amplified, rather than attenuated, the association between stress and negative affect. Overall, findings support robust momentary emotional benefits of interacting with companion animals, but do not support stress-buffering as the mechanism underlying this association. These results emphasize the importance of considering both species and situational context when examining the psychological impact of human–animal interaction.

## 1 Introduction

Companion animals play an integral role in human society, often regarded as family members that foster profound emotional connections (Allen, 2003; Herzog, 2011; Applebaum and Zsembik, 2020). It is widely believed that people derive benefits from their companionship, a phenomenon often referred to as the” pet-effect,” which describes the idea that pet ownership or interaction is associated with improvements in psychological wellbeing and reduced distress (Herzog, 2011). However, empirical findings on the pet-effect are mixed, with studies reporting positive, null, and even negative associations between HAI and mental health (e.g., Bao and Schreer, 2016; Parslow et al., 2005). These inconsistencies likely reflect methodological differences and the complex, multifaceted nature of human–animal relationships. The potential benefits of companion animals may depend on factors such as the type of interaction, and characteristics of both the owner and the animal (Herzog, 2011; Rodriguez et al., 2021). This heterogeneity appears not only between studies but also within individuals, as ecological momentary assessment (EMA) studies suggest that even within individuals the effects of animals are heterogeneous, for example the mere presence of an animal may reduce negative affect, whereas active interaction increases positive affect (Janssens et al., 2020; Scoresby et al., 2021). Together, these findings indicate that the pet-effect cannot be understood as a uniform phenomenon and that more research is needed to clarify the conditions under which interactions with animals influence wellbeing. In addition to questions about the consistency of the beneficial effects of companion animals on human wellbeing, the mechanisms underlying these potential benefits remain insufficiently understood. One frequently proposed explanation is the stress-buffering hypothesis (Serpell, 2016; Crossman, 2017). Animals might provide social and emotional support during stressful circumstances, potentially stemming from the perception of animals as part of one’s social network (McConnell et al., 2011). While several studies support these stress-buffering effects (Beetz et al., 2012; Rodriguez et al., 2021), findings remain context-dependent and may vary depending on stress assessment (Martins et al., 2023). Taken together, the mixed evidence for the beneficial effects of companion animals and the limited understanding of its underlying mechanisms highlight the need for more nuanced approaches to studying human-animal interaction. One factor that has received relatively little attention in this context is the species of the companion animal. Dogs and cats differ markedly in their behavioral characteristics and patterns of interaction with owners (González-Ramírez and Landero-Hernández, 2021), suggesting that their associations with emotional experiences and stress processes may also differ. Examining potential species differences may therefore provide valuable insight into when and how companion animals influence human affect and wellbeing. Accordingly this study investigates how interactions with dogs and cats may differ in their associations with affect and in the underlying mechanisms through which companion animals influence wellbeing. Specifically, it is hypothesized that interactions with dogs and cats differ in their associations with momentary positive and negative affect. We also expect that interacting with a companion animal reduces the detrimental effects of event-related and activity-related stress on positive and negative affect, and that this stress-buffering effect is stronger for dogs than for cats. By examining these species-specific patterns in affective experiences and stress-buffering processes, this research aims to advance our understanding of the mechanisms underlying the beneficial influence of companion animals on emotional wellbeing and its variability across companion animal species.

While research on human–animal interaction has grown more refined and nuanced in recent years, the beneficial influence of companion animals on emotional wellbeing and the mechanisms underlying these effects remain incompletely understood. Further investigation is warranted to determine their generalizability and specificity. To date, much of this refinement has focused on the human aspect of this interaction, with studies indicating that human-related factors such as gender, age and employment status may shape the nature and strength of the pet effect (Nimer and Lundahl, 2007; Miller et al., 2009; Amiot and Bastian, 2015; Mueller et al., 2021; Scoresby et al., 2021). In contrast, variability in the characteristics of the animals involved in HAI has received comparatively little attention. The most obvious factor in this regard is the animal’s species. Most studies are conducted within a single species, often dogs or cats, yet it has been suggested that species differences may also account for part of the heterogeneity observed across HAI research (Rodriguez et al., 2021).

To address this gap, the present study aims to explore whether the beneficial influence of companion animals on emotional wellbeing, as well as the mechanisms underlying this influence, are species-specific. We focus on dogs versus cats as these are two of the most prevalent companion animals. Dogs and cats are distinct species with different evolutionary backgrounds, which contribute to their unique behavioral and psychological attributes. Research indicates that different activities involving dogs affect human wellbeing differently, with closer relationships between owners and dogs amplifying the benefits. Positive interactions like tactile contact and walking have been associated with increased wellbeing, whereas negative events such as health problems or aggression can have the opposite effect (Barcelos et al., 2021). Matijczak et al. (2023) demonstrated that interacting with dogs, particularly after stressful events, can lead to immediate increases in positive emotions and reductions in anxiety, underscoring the important role of the human–dog bond. In the same study, stronger engagement with one’s dog predicted lower momentary negative affect (Matijczak et al., 2023). While the impact of HAI on wellbeing has been extensively documented in dogs, research on cats remains relatively limited. Positive interactions with cats, such as caregiving and tactile contact, may enhance wellbeing, but these effects are less frequently reported than for dogs (Delanoeije and Pendry, 2023). Negative experiences, such as failure to meet their needs or coping with problematic behaviors, can reduce wellbeing for both species (Ravenscroft et al., 2021; Ståhl et al., 2023).

Only a limited number of studies have directly compared the effects of dog and cat ownership on human wellbeing, yet the few that exist suggest notable differences between the two. Recent research suggests that dog owners generally report higher self-esteem compared to individuals without companion animals, whereas cat owners tend to have marginally lower self-esteem scores, particularly among women (Schulz et al., 2020). In a longitudinal study of 2,584 adolescents, Endo et al. (2020) found that dog ownership was associated with improved mental wellbeing, while cat ownership showed a negative association (Endo et al., 2020). Similarly, Bao and Schreer (2016) reported that dog owners were happier, more satisfied with life, and experienced more positive and fewer negative emotions than cat owners. However, these differences were partly attributable to underlying factors such as personality traits, emotion regulation, and basic need satisfaction (Bao and Schreer, 2016). This suggests that observed wellbeing differences between dog and cat owners are not solely driven by the species itself but may also reflect the characteristics of individuals who tend to choose a particular type of pet. The bond or relationship between human and companion animal also seems to differ somewhat between dogs and cats; emotional closeness tends to be higher with dogs (González-Ramírez and Landero-Hernández, 2021), possibly due to differences in engagement requirements and human perceptions of the pets’ needs (Ståhl et al., 2023; Westgarth et al., 2019). Consequently, the dynamics of the human–animal relationship, and their impact on psychological outcomes, reflect a combination of species-specific behaviors and human social beliefs (Rodriguez et al., 2021). Building on these insights, the present study tests whether interactions with dogs and cats differ in their associations with momentary positive and negative affect, and whether these associations are moderated by stress.

## 2 Materials and methods

### 2.1 Design

This research adopts an observational approach and comprises two components: (1) a survey employing a cross-sectional design and (2) an EMA study employing an intensive longitudinal design.

### 2.2 Participants

Respondents were recruited through selective sampling, with predefined inclusion criteria ensuring eligibility for participation. Participants had to be at least 18 years old, reside in the Netherlands or Belgium, own at least one dog or one cat, and possess a functional smartphone. Recruitment was conducted by graduate and undergraduate students of the Open University of the Netherlands, utilizing personal networks, social media, veterinary practices, pet stores, and pet owner clubs/associations. Participation was voluntary and anonymous.

### 2.3 Procedure

This study was part of a larger research project examining various aspects of human–animal interactions, including specific characteristics of companion animals and their owners which was published as a study protocol (Janssens et al., 2024). For the current study, only the information relevant to animal interaction, affect, and stress was included and reported. The procedure for this study involved a multi-step approach to ensure comprehensive data collection from pet owners. Pet owners interested in participating were directed to the study website, where they received detailed information about the study, the procedures involved, and additional instructions on completing the EMA. Upon participation, they proceeded to LimeSurvey (2022) to provide online informed consent and complete an online questionnaire. This questionnaire gathered demographic characteristics of the owners and detailed information about their pet ownership and pet’s characteristics.

After completing the questionnaires, participants were instructed to download the RealLife Exp (Lifedata, 2021) app on their smartphones. They then entered the password that was sent via email to ensure the correct linkage of data from the questionnaires to the app.

Data collection through the app commenced the following day, with participants receiving ten notifications per day over five consecutive days, at semi-random moments between 07:30 and 22:30. The study design ensured the inclusion of non-working days to increase the likelihood of pet presence and was conducted during a typical workweek to avoid holidays or special events. The notifications prompted participants to complete a short questionnaire on their smartphones, capturing their current emotion, social context, activities, location, and interactions with their companion animal. To minimize memory distortion, participants were encouraged to complete the questionnaire promptly after receiving the notification, with the questionnaire expiring after fifteen minutes. This high-density signaling approach, previously validated in similar studies (Hektner et al., 2007; Janssens et al., 2020, 2021), ensured reliable and valid data collection.

The study was approved by the Research Ethics Committee (cETO) of the Open University, Netherlands (U202208386).

### 2.4 Materials

#### 2.4.1 Demographics

The demographic questionnaire administered in this study encompasses a range of variables pertaining to both the owner and their companion animal. Pet owner characteristics include age, gender, educational attainment, marital status, and household composition. Additionally, the species of the companion animal was collected.

#### 2.4.2 EMA questionnaire

The EMA questions used in this study focused on participants’ mood, ongoing activities, and the presence or interaction with their companion animal at the moment of reporting. The analyses included the following measures:

Momentary affect: Participants’ affect was assessed using mood-related adjectives adapted from the Positive and Negative Affect Schedule (Crawford and Henry, 2004). The selected items were those previously shown to load highly on latent factors of positive and negative affect and to exhibit sufficient within-person variability in EMA studies (Jacobs et al., 2007; Janssens et al., 2020; Wichers et al., 2007). The items capture a broad range of affective states across both valence (positive–negative) and arousal (high–low) dimensions (Kuppens et al., 2013). Positive affect was computed as the mean rating on items reflecting cheerful, satisfied, happy, and enthusiastic affect, while negative affect was based on items capturing feelings of insecurity, loneliness, anxiety, irritation, sadness, and guilt. Questions were formulated as “at this moment, I feel…” and responses were given on a 7-point Likert scale ranging from 1 (“not at all”) to 7 (“very”).

Momentary stress: Stress was operationalized as the subjective appraisal of the current activity or significant events (Vaessen et al., 2016). Activity-related stress was assessed with three statements about the ongoing activity: “I would rather do something else,” “This takes effort,” and “I am good at this,” rated on a 7-point Likert scale (1 = not at all, 7 = very). Activity-related stress was computed as the mean score across the three items, with the item “I am good at this” reverse-coded such that higher scores indicate higher levels of activity-related stress. Event-related stress was measured by asking participants to rate the most important event since the previous notification on a bipolar scale from −3 (“very unpleasant”) to +3 (“very pleasant”). In line with transactional stress theory (Lazarus and Folkman, 1984) and prior EMA research (e.g., Janssens et al., 2021; van Knippenberg et al., 2018), stress was conceptualized as arising from negatively appraised events involving perceived harm, loss, or threat. Therefore, positive values were recoded to 0 (no stress), and higher values reflected higher stress after reverse-coding. This operationalization isolates negatively appraised event-related stress from general affective valence.

Pet interaction: Interaction with the companion animal was assessed only when the animal was present, using the item “We are interacting,” rated on a 7-point scale (1 = not at all, 7 = very), with higher scores indicating more interaction.

Animal species: When a companion animal was present at the moment of the notification, participants indicated whether it was a cat, a dog, or both. Cases in which both a cat and a dog were present at that moment were excluded from the analyses, as these did not allow for clear comparisons between species.

### 2.5 Analyses

All analyses were performed in RStudio (version 2024.09.1). As EMA data have a hierarchical structure, with repeated measurements (level 1) nested within individuals (level 2), multilevel regression models were used to analyze the data. All models accounted for serial dependency, allowing residuals to be correlated over time [AR(1)], and included random intercepts and slopes across individuals.

All level 1 independent variables (momentary pet interaction, event-related stress, and activity-related stress) were person-mean centered such that each observation represents a deviation from that individual’s average, allowing coefficients to be interpreted as the effect of momentary deviations from an individual’s mean level. Distributions of the continuous EMA variables were examined using histograms, Q-Q plots, and Shapiro-Wilk tests, which indicated some non-normality and occasional extreme values. These features are common in EMA data, reflecting real-world variability. Linear mixed-effects models with random effects and AR(1) residual correlations are robust to such non-normality and extreme observations, and therefore no transformations or outlier removal were applied (Bolger and Laurenceau, 2013).

Age and gender, collected via the cross-sectional survey, were included as stable covariates, while companion animal species (reflected in the presence of a dog vs. cat) was included as a grouping factor to examine potential species-specific differences in the associations between level 1 predictors and momentary affect. Human presence at the moment of reporting (reflected in the variable ‘being alone’) was included as a time-varying covariate. Additional demographic characteristics, marital status, educational level, living situation and employment status, were reported descriptively to characterize the sample.

All available EMA observations were included in the analyses to avoid bias associated with arbitrary compliance thresholds (Rintala et al., 2019; Wrzus and Neubauer, 2023). Therefore, the number of observations contributing to each analysis may vary depending on data completeness for the variables of interest.

Prior to hypothesis testing, descriptive analyses were conducted to characterize the sample and the measured variables. Reliability analyses were performed on the affect scales to ensure internal consistency. Because two parallel models were estimated for the affective outcomes (positive affect and negative affect), each testing the same set of hypotheses, a Bonferroni correction was applied at the level of the dependent variables. This resulted in a corrected significance threshold of *p* < 0.025 for each model. Effects were considered significant if *p*-values were below this threshold. Covariates were not included in the correction, and their *p*-values are reported as observed.

The analyses followed a hierarchical structure aligned with the two central research questions. First, to examine whether the daily-life pet effect could be replicated (Janssens et al., 2020) and whether this effect differed between dog and cat owners, models included the two-way interaction between pet interaction and pet type (dog vs. cat), together with the corresponding main effects and covariates.

Second, to assess whether pet interaction functioned as a buffer in stressful situations, and whether this pattern differed between dogs and cats, models additionally included the three-way interaction between pet interaction, stress (event or activity-related), and pet type, along with all lower-order interaction terms and covariates.

Interactions were evaluated in a stepwise manner. When the three-way interaction was significant, follow-up analyses examined the simple two-way interactions between pet interaction and stress separately for dog owners and cat owners. When the three-way interaction was not significant, the corresponding two-way interaction between pet interaction and stress was interpreted. Main effects of pet interaction were only examined when no higher-order interactions involving this variable were present. Effect sizes and 95% confidence intervals were computed for all models.

## 3 Results

### 3.1 Descriptive statistics and reliability analyses

A total of 188 participants were recruited for the study, providing 7,963 EMA notifications. EMA compliance varied across participants (mean = 32.4%, SD = 20.9%, range = 0–86%). Reliability analyses were conducted on the affect scales. The negative affect scale, consisting of six items, demonstrated good reliability with α(aggregated) = 0.91 and α(within) = 0.68. The positive affect scale, comprising four items, showed excellent reliability with α(aggregated) = 0.96 and α(within) = 0.83. Descriptive statistics are provided in Table 1.

**TABLE 1:** Descriptive statistics of sample.

Participant-level variables	*n*	%	Range	*M*	*SD*
Age			19–79	44.3	13.9
Gender					
Male	42	22.3			
Female	146	77.7			
Marital status					
Single	27	14.4			
In a relationship, not living together	15	8.0			
Married/living together	138	73.4			
Divorced	4	2.1			
Widowed	4	2.1			
Highest educational level					
No education	0	0			
Primary education	0	0			
Lower vocational education	0	0			
Intermediate secondary education	11	5.9			
Higher secondary education	11	5.9			
Pre-university education	7	3.7			
Intermediate vocational education	39	20.7			
Higher vocational education	72	38.3			
Scientific education (university)	48	25.5			
Living situation					
Alone	34	18.1			
With parent(s)	11	5.9			
With partner/family	139	73.9			
Other	4	2.1			
Employment status					
Unemployed	35	18.6			
School/education	13	6.91			
Regular full-time job (from 32 h)	58	30.8			
Regular part-time job (up to 32 h)	82	43.6			
Companion animal					
Dog	75	60.5			
Cat	36	29.0			
Both	13	10.5			
Notification-level variables					
Positive affect	4433	43.2	1–7	4.76	1.37
Negative affect	4432		1–7	1.48	0.76
Event stress	4394		0–3	0.22	0.64
Activity stress	4418		1–7	2.65	1.38
Being alone	1907				
Dog present	1682	61.8			
Cat present	719	26.4			

*N* = 7963 notifications from 188 participants, *M*, mean; *SD*, standard deviation. Descriptive statistics for continuous variables (positive affect, negative affect, event stress, activity stress) are based on valid responses per variable (range: *n* = 4394–4433). Being alone is based on valid responses to the momentary social context item (*n* = 4413). The percentage reflects the proportion of notifications in which participants reported being alone. Pet presence variables are based on valid responses to the conditional ESM item assessing whether (only) a dog or cat is present during the notification. This item was only available when participants indicated pet presence earlier in the questionnaire, resulting in a reduced valid sample (*n* = 2723). Percentages are calculated within this subset.

All models were controlled for age, gender, and being alone. Covariates are only reported below when statistically significant.

### 3.2 Companion animals’ contribution to emotional wellbeing in daily life

#### 3.2.1 Interaction between pet interaction and pet type on positive affect and negative affect

The interaction term between pet interaction and pet type (dog vs. cat) was not significant for either positive affect (*B* = −0.04, *p* = 0.053, CI [−0.09, 0.00]) or negative affect (*B* = −0.01, *p* = 0.326, CI [−0.03, 0.01]). These findings indicate that pet type did not moderate the association between pet interaction and momentary affect. Contrary to our hypothesis, the expected stronger effect for dogs was not observed. None of the covariates reached significance in these models (Table 2).

**TABLE 2:** Mixed-effects model between pet interaction and pet type on positive affect and negative affect.

Outcome	Variable	*B*	SE	95% CI	*t*	*P*
Positive affect	Age	0.01	0.01	[−0.00, 0.02]	1.50	0.136
	Gender	−0.14	0.19	[−0.51, 0.23]	−0.76	0.447
	Alone	−0.14	0.05	[−0.23, −0.05]	−3.11	0.002
	Pet interaction	0.08	0.01	[0.06, 0.11]	6.81	0.000
	Pet type	−0.14	0.16	[−0.45, 0.16]	−0.90	0.368
	Pet interaction × pet type	−0.04	0.02	[−0.09, 0.00]	−1.93	0.053
Negative affect	Age	−0.01	0.00	[−0.01, 0.00]	−1.83	0.069
	Gender	−0.22	0.12	[−0.46, 0.02]	−1.89	0.061
	Alone	0.05	0.03	[−0.01, 0.08]	1.81	0.071
	Pet interaction	−0.02	0.01	[−0.03, −0.01]	−3.17	0.002
	Pet type	0.01	0.09	[−0.18, 0.18]	0.08	0.940
	Pet interaction × pet type	−0.01	0.01	[−0.03, 0.01]	−1.00	0.326

*N* = 2385 observations from 169 participants. Gender: 0 = male, 1 = female; pet type: 0 = dog, 1 = cat; Alone: 0 = no, 1 = yes. B, unstandardized coefficient; SE, standard error; 95% CI, 95% confidence interval; *t*, *t*-value; *p*, *p*-value. Significance threshold: α = 0.025.

#### 3.2.2 Main effects of pet interaction on positive affect and negative affect

Because no significant interaction between pet interaction and pet type was found, the main effects of pet interaction were examined to assess the overall association between interacting with companion animals and emotional wellbeing. Pet interaction was significantly associated with increased positive affect (*B* = 0.06, *p* < 0.001, CI [0.04, 0.08]), showing that moments in which the level of interaction with a companion animal is higher, individuals experience more positive affect. Being alone was significantly associated with lower positive affect (*B* = −0.15, *p* < 0.001, CI [−0.24, −0.06]).

Also, pet interaction was significantly associated with lowered negative affect (*B* = −0.02, *p* < 0.001, CI [−0.03, −0.01]), showing that moments in which the level of interaction with a companion animal is higher negative affect is lower. Being alone was significantly associated with higher negative affect (*B* = 0.05, *p* = 0.019, CI [0.01, 0.09]) (Table 3).

**TABLE 3:** Main effects of interaction with a companion animal with positive affect and negative affect.

Outcome	Variable	B	SE	95% CI	*t*	*p*
Positive affect	Age	0.01	0.01	[−0.00, 0.02]	1.73	0.085
	Gender	−0.21	0.18	[−0.56, 0.14]	−1.15	0.252
	Alone	−0.15	0.04	[−0.24, −0.06]	−3.55	0.000
	Pet interaction	0.06	0.01	[0.04, 0.08]	6.47	0.000
Negative affect	Age	−0.01	0.00	[−0.01, 0.00]	−1.95	0.053
	Gender	−0.22	0.11	[−0.44, 0.00]	−1.97	0.050
	Alone	0.05	0.02	[0.01, 0.09]	2.35	0.019
	Pet interaction	−0.02	0.00	[−0.03, −0.01]	−4.83	0.000

*N* = 2707 observations from 171 participants. Gender: 0 = male, 1 = female; pet type: 0 = dog, 1 = cat; Alone: 0 = no, 1 = yes. B, unstandardized coefficient; SE, standard error; 95% CI, 95% confidence interval; *t*, *t*-value; *p*, *p*-value. Significance threshold: α = 0.025.

### 3.3 Stress-buffering effects

#### 3.3.1 Three-way interaction: event stress, pet interaction, and pet type

To examine whether stress-buffering patterns would emerge for activity-related stress, we next tested the three-way interaction between event stress, pet interaction, and pet type. Contrary to our hypothesis, the interaction was not significant for positive affect (*B* = −0.06, *p* = 0.040, CI [−0.12, 0.00]) at the adjusted α of 0.025, suggesting no significant moderating effect of pet type on the interaction between pet interaction and event stress for positive affect. Being alone was significantly associated with lower positive affect (*B* = −0.10, *p* = 0.016, CI [−0.18, −0.02]), while age and gender were not significant covariates.

For negative affect, the three-way interaction between pet type, pet interaction, and event stress was significant (*B* = 0.04, *p* = 0.007, CI [0.01, 0.07]), indicating that the association between event stress and negative affect varied depending on whether participants interacted with a dog or a cat. However, the effect size indicates a stronger association for cats compared to dogs. This is contrary to the hypothesis predicting stronger stress-buffering for dogs. Among the covariates, none reached significance (Table 4).

**TABLE 4:** Three-way interaction between pet interaction, event stress and pet type (dog vs. cat) for positive affect and negative affect.

Outcome	Predictor	B	SE	95% CI	*t*	*p*
Positive affect	Age	0.01	0.01	[−0.00 to 0.01]	1.46	0.146
	Gender	−0.14	0.19	[−0.51 to 0.23]	−0.74	0.458
	Alone	−0.10	0.04	[−0.18 to −0.02]	−2.41	0.016
	Pet interaction	0.08	0.01	[0.06 to 0.10]	7.21	0.000
	Event stress	−0.36	0.04	[−0.43 to −0.28]	−9.48	0.000
	Pet type	−0.14	0.15	[−0.44 to 0.16]	−0.88	0.379
	Pet interaction × event stress	0.04	0.02	[0.01 to 0.08]	2.37	0.018
	Pet interaction × pet type	−0.05	0.02	[−0.09 to-0.01]	−2.55	0.011
	Event stress × pet type	−0.20	0.06	[−0.32 to −0.08]	−3.36	0.001
	Pet interaction × event stress × pet type	−0.06	0.03	[−0.12 to 0.00]	−2.06	0.040
Negative affect	Age	−0.01	0.00	[−0.01 to 0.00]	−1.81	0.072
	Gender	−0.22	0.12	[−0.45 to −0.01]	−1.94	0.054
	Alone	0.03	0.02	[−0.02 to 0.07]	1.18	0.238
	Pet interaction	−0.01	0.01	[−0.02 to −0.01]	−2.79	0.005
	Event stress	0.14	0.02	[0.10 to 0.18]	6.72	0.000
	Pet type	0.00	0.09	[−0.17 to 0.18]	−0.01	0.993
	Pet interaction × event stress	−0.00	0.01	[−0.02 to 0.01]	−0.42	0.675
	Pet interaction × pet type	−0.01	0.01	[−0.02 to 0.01]	−0.34	0.732
	Event stress × pet type	0.13	0.03	[0.06 to 0.20]	4.06	0.000
	Pet interaction × event stress × pet type	0.04	0.02	[0.01 to 0.07]	2.72	0.007

*N* = 2385 observations from 169 participants. Gender: 0 = male, 1 = female; pet type: 0 = dog, 1 = cat; Alone: 0 = no, 1 = yes. B, unstandardized coefficient; SE, standard error; 95% CI, 95% confidence interval; *t*, *t*-value; *p*, *p*-value. Significance threshold: α = 0.025.

#### 3.3.2 Interaction between pet interaction and event stress on positive affect

Since the three-way interaction was not significant for positive affect, we examined the overall two-way interaction between pet interaction and event-related stress in this model. This interaction was not significant for positive affect (*B* = 0.01, *p* = 0.315, CI [−0.01, 0.04]), indicating that the association between event stress levels and affect was not contingent on pet interaction. Among the covariates, age was significantly associated with higher positive affect (*B* = 0.01, *p* = 0.012, CI [0.00, 0.03]), and being alone was significantly associated with lower positive affect (*B* = −0.11, *p* = 0.005, CI [−0.19, −0.03]). Gender was not significantly related to positive affect (Table 5).

**TABLE 5:** Interaction between pet interaction and event stress for positive affect.

Outcome	Variable	*B*	SE	95% CI	*T*	*p*
Positive affect	Age	0.01	0.01	[0.00 to 0.03]	2.55	0.012
	Gender	−0.26	0.19	[−0.63 to 0.10]	−1.40	0.162
	Alone	−0.11	0.04	[−0.19 to −0.03]	−2.83	0.005
	Pet interaction	0.05	0.01	[0.04 to 0.07]	6.88	0.000
	Event stress	−0.41	0.04	[−0.50 to −0.33]	−9.27	0.000
	Pet interaction × event stress	0.01	0.01	[−0.01 to 0.04]	1.00	0.315

*N* = 2707 observations from 171 participants. Gender: 0 = male, 1 = female; pet type: 0 = dog, 1 = cat; Alone: 0 = no, 1 = yes. B, unstandardized coefficient; SE, standard error; 95% CI, 95% confidence interval; *t*, *t*-value; *p*, *p*-value. Significance threshold: α = 0.025.

#### 3.3.3 Species specific interactions between event stress and pet interaction on negative affect

To further explore the significant three-way interaction for negative affect, separate models were estimated for dogs and cats.

##### 3.3.3.1 Dogs

For the dog sample (*n* = 1,667 observations, 118 participants), the interaction between pet interaction and event stress was not significant (*B* = −0.00, *p* = 0.735, CI [−0.02, 0.02]), indicating that interaction with a dog did not moderate the association between event stress and negative affect. This pattern is inconsistent with the hypothesis that dog interaction would reduce negative affect under stress. Among the covariates, only pet interaction was significantly associated with lower negative affect (*B* = −0.01, *p* = 0.005, CI [−0.02, 0.00]), while age, gender, and being alone were not significant (Table 6).

**TABLE 6:** Pet interaction and event stress in relation to negative affect for dogs.

Outcome	Variable	B	SE	95% CI	*t*	*p*
Negative affect	Age	−0.01	0.00	[−0.01, 0.00]	−1.35	0.181
	Gender	−0.23	0.14	[−0.50, 0.04]	−1.68	0.096
	Alone	0.02	0.03	[−0.03, 0.07]	0.58	0.564
	Pet interaction	−0.01	0.00	[−0.02, 0.00]	−2.84	0.005
	Event stress	0.14	0.02	[0.10, 0.18]	7.15	0.000
	Pet interaction × event stress	−0.00	0.01	[−0.02, 0.02]	−0.34	0.735

*N* = 1667 observations from 118 participants. Gender: 0 = male, 1 = female; Alone: 0 = no, 1 = yes. *B*, unstandardized coefficient; SE, standard error; 95% CI, 95% confidence interval; *t*, *t*-value; *p*, *p*-value. Significance threshold: α = 0.05.

##### 3.3.3.2 Cats

For the cat sample (*n* = 718 observations, 58 participants), the interaction between pet interaction and event stress was significant (*B* = 0.04, *p* = 0.007, CI [0.01, 0.07]), indicating that interaction with cats moderated the association between event stress and negative affect. This indicates that negative affect increases more strongly under stress when participants show a higher level of interaction with their cat. This finding contrasts with the hypothesis that stress-buffering effects would be stronger for dogs compared to cats. None of the covariates were significantly associated with negative affect in the cat model (Table 7).

**TABLE 7:** Pet interaction and event stress in relation to negative affect for cats.

Outcome	Variable	B	SE	95% CI	*t*	*p*
Negative affect	Age	−0.01	0.01	[−0.02, 0.01]	−0.77	0.444
	Gender	−0.23	0.22	[−0.66, 0.20]	−1.04	0.305
	Alone	0.07	0.06	[−0.04, 0.18]	1.23	0.218
	Pet interaction	−0.02	0.01	[−0.04, 0.00]	−2.07	0.039
	Event stress	0.24	0.03	[0.19, 0.30]	8.69	0.000
	Pet interaction × event stress	0.04	0.01	[0.01, 0.07]	2.72	0.007

*N* = 718 observations from 58 participants. Gender: 0 = male, 1 = female; Alone: 0 = no, 1 = yes. B, unstandardized coefficient; SE, standard error; 95% CI, 95% confidence interval; *t*, *t*-value; *p*, *p*-value. Significance threshold: α = 0.05.

#### 3.3.4 Three-way interaction: activity stress, pet interaction, and pet type

To examine whether the type of animal moderates the potential stress-buffering effect of interacting with a companion animal for activity related stress, three-way interactions between pet type (dog/cat), pet interaction, and activity stress were tested. These interactions were not significant for either positive affect (*B* = 0.02, *p* = 0.115, CI [−0.01, 0.05]) or negative affect (*B* = −0.01, *p* = 0.187, CI [−0.03, −0.01]), providing no evidence for the hypothesized stress-buffering effect of companion animal interaction during activity-related stress. None of the covariates reached significance for positive affect or negative affect at the α = 0.025 threshold (Table 8).

**TABLE 8:** Three-way interaction between pet interaction, activity stress and pet type (dog vs. cat) for positive affect and negative affect.

Outcome	Variable	B	SE	95% CI	*t*	*p*
Positive affect	Age	0.01	0.01	[0.00, 0.02]	2.19	0.030
	Gender	−0.18	0.19	[−0.54, 0.18]	−0.94	0.347
	Alone	−0.05	0.04	[−0.13, 0.03]	−1.35	0.176
	Pet interaction	0.04	0.01	[0.02, 0.06	3.67	0.000
	Activity stress	−0.37	0.02	[−0.41, −0.33]	−20.33	0.000
	Pet type	−0.19	0.15	[−0.49, 0.10]	−1.29	0.197
	Pet interaction × activity stress	−0.01	0.01	[−0.03, 0.01]	−1.56	0.119
	Pet interaction × pet type	−0.01	0.02	[−0.05, 0.03]	−0.62	0.532
	Activity stress × pet type	0.00	0.03	[−0.06, 0.06]	0.01	0.993
	Pet interaction × activity stress × pet type	0.02	0.01	[−0.01, 0.05]	1.58	0.115
Negative affect	Age	−0.01	0.00	[−0.01, 0.00]	−1.99	0.048
	Gender	−0.22	0.12	[−0.45, 0.01]	−1.99	0.060
	Alone	0.00	0.02	[−0.04, 0.05]	0.19	0.847
	Pet interaction	0.00	0.01	[−0.04, 0.05]	0.03	0.976
	Pet type	0.01	0.09	[−0.16, 0.18]	0.15	0.882
	Activity stress	0.15	0.01	[0.13, 0.17]	14.54	0.000
	Pet interaction × activity stress	0.00	0.00	[−0.01, 0.01]	0.80	0.425
	Pet interaction × pet type	−0.02	0.01	[−0.04, 0.00]	−2.10	0.036
	Activity stress × pet type	0.04	0.02	[0.00, 0.07]	2.14	0.032
	Pet interaction × activity stress × pet type	−0.01	0.01	[−0.03, −0.01]	−1.32	0.187

*N* = 2,385 observations from 169 participants. Gender: 0 = male, 1 = female; pet type: 0 = dog, 1 = cat; Alone: 0 = no, 1 = yes. B, unstandardized coefficient; SE, standard error; 95% CI, 95% confidence interval; *t*, *t*-value; *p*, *p*-value. Significance threshold: α = 0.025.

#### 3.3.5 Interaction between pet interaction and activity stress on positive affect and negative effect

Since the three-way interaction was not significant, follow-up two-way interaction models were estimated to test whether a general stress-buffering effect of interacting with a companion animal was present. The interaction between pet interaction and activity stress was not significant for either positive affect (*B* = 0.00, *p* = 0.498, CI [−0.02, 0.01]) or negative affect (*B* = 0.00, *p* = 0.881, CI [−0.01, 0.01]). This indicates that the association between activity stress and momentary affect was not contingent on interaction with the companion animal. Among the covariates, age was positively associated with positive affect (*B* = 0.02, *p* = 0.007, CI [0.00, 0.03]). Neither gender nor being alone was significantly associated with momentary affect at the α = 0.025 threshold in these models (Table 9).

**TABLE 9:** Two-way interaction between pet interaction and activity stress.

Outcome	Variable	B	SE	95% CI	*t*	*p*
Positive affect	Age	0.02	0.01	[0.00 to 0.03]	2.72	0.007
	Gender	−0.22	0.18	[−0.57 to 0.13]	−1.23	0.219
	Alone	−0.04	0.04	[−0.11 to −0.03]	−0.96	0.338
	Pet interaction	0.02	0.01	[0.01 to 0.04	3.47]	0.001
	Activity stress	−0.35	0.02	[−0.37 to −0.31]	−15.73	0.000
	Pet interaction × activity stress	0.00	0.01	[−0.02 to 0.01]	−0.68	0.498
Negative affect	Age	−0.01	0.00	[−0.01 to 0.00]	−2.11	0.036
	Gender	−0.22	0.11	[−0.44 to 0.00]	−1.97	0.050
	Alone	0.01	0.02	[−0.04 to 0.06]	0.38	0.703
	Pet interaction	−0.01	0.00	[−0.01 to 0.00]	−1.45	.0148
	Activity stress	0.16	0.01	[0.14 to 0.18]	20.10	0.000
	Pet interaction × activity stress	0.00	0.00	[−0.01 to 0.01]	−0.15	0.881

*N* = 2,707 observations from 171 participants. Gender: 0 = male, 1 = female; pet type: 0 = dog, 1 = cat; Alone: 0 = no, 1 = yes. B, unstandardized coefficient; SE, standard error; 95% CI, 95% confidence interval; *t*, *t*-value; *p*, *p*-value. Significance threshold: α = 0.025.

## 4 Discussion

The present study examined the association between interaction with a companion animal and momentary emotional wellbeing of pet owners, as measured by positive affect and negative affect, and investigated whether companion animals buffer the impact of stress on affect. Building on the idea that the effects of HAI may differ across species, we specifically explored whether interactions with dogs and cats vary in their associations with human affective experiences, and whether these interactions buffer the impact of both event-related and activity-related stress differentially. By examining these species-specific patterns, the study aimed to shed light on the mechanisms through which companion animals influence emotional wellbeing in daily life.

### 4.1 Companion animals’ contribution to emotional wellbeing in daily life

The present findings showed that a higher level of interaction with a companion animal was associated with increased positive affect and decreased negative affect, confirming that companion animals contribute to momentary emotional wellbeing. These associations remained significant even after controlling for the potential confounders, human presence, age of the pet owners, and gender of the pet owners. Notably, the measure of social context only distinguished between being alone or being with other humans, without capturing the quality or closeness of human interactions. As a result, we cannot conclude whether the benefits of animal interaction are independent of support from close humans. Nonetheless, these findings are consistent with prior research indicating that interactions with companion animals can enhance momentary emotional wellbeing (Handlin et al., 2011; Beetz et al., 2012; Janssens et al., 2020).

Contrary to some previous suggestions that dogs may offer stronger emotional benefits due to their social and affiliative nature (Powell et al., 2019), the interaction between interacting with a companion animal and animal species was not significant for either positive affect or negative affect. This suggests that the positive effects of animal interaction are not species-specific, and that cats can provide emotional comfort and support comparable to that provided by dogs, albeit potentially through different interaction styles (Turner, 2017). For example, it is plausible that dogs may enhance emotional wellbeing through active social engagement and responsiveness, whereas cats may provide comfort via more passive companionship and consistent presence (McNicholas and Collis, 2000; Ines et al., 2021; Turner, 2021). Although in the present study the overall emotional benefits appear comparable across species, the mechanisms through which these benefits are realized may differ, reflecting differences in interaction styles and owner expectations. The lack of species differences, as found in the present study, also aligns with prior work indicating inconsistent effects of type of species on psychological outcomes including perceived stress, mood, and broader mental health indicators (Cohen et al., 2007; Wells, 2009). Taken together, these results provide evidence that engaging with a companion animal, whether dog or cat, is associated with improvements in momentary emotional wellbeing.

### 4.2 Stress-buffering by companion animals

Having established that interactions with companion animals are associated with enhanced momentary emotional wellbeing, we next examined whether such interactions can also buffer the negative impact of stress on affect, focusing on both event-related and activity-related stress, and considering potential differences between dogs and cats. Overall, no evidence was found for a general stress-buffering effect of companion animals. Neither the interaction with a dog as the interaction with a cat buffered against the detrimental effects of activity or event related stress on positive and negative affect. Interestingly, the only significant effect was species-specific and emerged in cats. It showed a moderating effect of interaction with cats on the association between event-related stress and negative affect, but not in the expected direction: interactions with cats did not attenuate the association between stress and negative affect but amplified this relationship. The moderation by interaction with cats thus reflects an increase in the affective reactivity to event related stress rather than the buffering of this response.

The absence of stress-buffering effects across all models calls for an explanation of why interactions with companion animals did not reduce the emotional impact of daily stress in the present study. As expected, daily-life stressful events and activities were associated with affective states, such that higher levels of stress coincided with lower positive affect and higher negative affect. A higher level of interaction with the companion animal however did not buffer against the negative impact of stress on affect. This suggests that stress-buffering is unlikely to be the mechanism underlying the association between interaction with the companion animal and affect in daily life. Importantly, the absence of a stress-buffering interaction in the present analyses does not necessarily imply that stress buffering is not an underlying mechanism in the broader association between companion animals and wellbeing. In the current study, analyses focused specifically on moments of interaction with the companion animal, which by definition implies that the animal was present. Moments in which the animal was not present were therefore not included in the analyses. As a result, the effects observed here should be interpreted as effects above and beyond the potential baseline influence of the animal’s presence itself. It is therefore possible that a stress-buffering effect would emerge when comparing moments in which the companion animal is present with moments in which it is absent. This explanation is consistent with earlier findings suggesting that stress-buffering effects may be linked primarily to the presence of the companion animal, rather than to additional interaction with the animal (Janssens et al., 2021). The decision to focus on interaction rather than mere presence was made for methodological reasons. Specifically, it allowed us to examine differences between interactions with dogs and cats while including owners who lived with both species, as the analyses were based on momentary interactions rather than on ownership of a particular animal type. When considered in the broader context of the present findings, however, the results still provide an important insight. Although interaction with the companion animal was associated with more positive affective states, it did not moderate the relationship between stress and affect. This suggests that the positive association between interacting with companion animals and wellbeing is unlikely to operate through a stress-buffering mechanism, at least at the momentary level examined in this study. Instead, the findings support the notion that multiple mechanisms may underlie the beneficial effects of companion animals on human wellbeing and is consistent with the broader literature on companion animals and stress, which reports heterogeneous and context-dependent findings (Rodriguez et al., 2021; Northrope et al., 2025).

The findings involving cats were somewhat unexpected and therefore warrant additional consideration. Whereas no comparable moderation effect emerged for dogs, interacting with cats was associated with a stronger link between event-related stress and negative affect. Given that this pattern runs counter to the more commonly assumed beneficial role of companion animals in coping with stress (Allen, Blascovich, and Mendes, 2002; Beetz et al., 2012), it is important to consider possible explanatory mechanisms. The cat-specific amplification effect for negative affect in response to event-related stress might possible be explained by psychological and relational mechanisms. Unlike dogs, cats often provide passive, low-demand companionship, which may make their interactions more salient or emotionally evocative under stressful circumstances, potentially amplifying stress responses when owners are already experiencing negative affect (Turner, 2021; Wells, 2009). This aligns with the broader social support literature showing that support can sometimes exacerbate stress when it is mismatched with the individual’s needs or the type of stressor, a phenomenon referred to as “support deterioration” (Bolger and Amarel, 2007; Seidman et al., 2006). In contrast, interactions with dogs often involve physical activity or social engagement (Barcelos et al., 2021; Wood et al., 2005), which may not coincide with momentary stress experiences in the same way, potentially explaining why no analogous amplification effect was observed for dogs in the present study. Although mechanisms of emotional attunement between humans and cats are not widely studied, existing research suggests that interactions with cats can produce immediate affective and physiological responses, including changes in current mood and physiological measures such as heart rate (Turner, 2021), which may increase the salience of stressful events and contribute to the stronger stress-affect coupling observed in the tested event-related stress model. An important note is that the observed interaction effect was relatively small in magnitude. Although small effects are common in experience sampling research and may still be meaningful in the context of momentary fluctuations in daily life (Myin-Germeys et al., 2009; Delespaul, 1995; Bolger and Laurenceau, 2013), the present finding should be interpreted with some caution. It is possible that the observed pattern reflects a subtle association rather than a robust effect, and future research will be needed to determine whether this finding replicates.

### 4.3 Strengths, limitations, and future directions

This study has several notable strengths. The use of EMA allowed for the real-time measurement of affective states alongside daily-life interactions with companion animals, thereby minimizing retrospective bias and reducing errors associated with recall (Scollon et al., 2003; Shiffman et al., 2008; Trull and Ebner-Priemer, 2013). The implicit nature of this approach enabled us to examine the influence of companion animals on affect without relying on participants’ cognitive interpretations, providing a more ecologically valid representation of HAI. Repeated assessments over time allowed each participant to serve as their own control, helping to account for pre-existing differences between pet owners. Additionally, EMA captured minor daily hassles and naturally occurring stressors, which are often overlooked in retrospective designs, allowing for a nuanced understanding of how companion animals may influence emotional responses to everyday stress.

Despite these strengths, several limitations should be noted. First, the cross-sectional component of the study precludes causal conclusions regarding the relationship between interaction with companion animals and emotional wellbeing. While the findings indicate that interactions with a companion animal are associated with momentary affect, time-lagged EMA analyses are needed to clarify the directionality of these effects and how long they persist. Second, participants were recruited using a convenience sampling strategy, facilitated by students from the Open University. Although convenience sampling can introduce bias due to self-selection and recruitment within specific social networks, given that Open University students are often older, employed, and have diverse life experiences, the sample recruited in their direct environment included adults with varied ages, work experience, and life circumstances. Nonetheless, recruitment through personal and social networks, together with the EMA requirements (e.g., smartphone ownership, willingness to respond to frequent prompts), may still limit generalizability (Bornstein et al., 2013). Third, the overall compliance rate in the present EMA study was somewhat lower than in studies that apply a minimum completion threshold. In this study, however, we intentionally retained all available data rather than excluding participants based on compliance criteria, meaning the reported compliance reflects the full variability in participant engagement. Importantly, there is no clear reason to assume that responses from participants with lower compliance are less reliable; they simply contribute fewer observations to the multilevel analyses. Fourth, the sample size for cat owners was smaller than that for dog owners, which may have limited statistical power for detecting other potential species-specific interactions. Although the amplification effect for cat owners was significant, the smaller sample size for this group may limit the generalizability of this finding and warrants replication in larger, balanced samples. In addition, we did not account for breed-specific or temperament-related differences among companion animals. Traits such as activity level, sociability, or responsiveness common to certain breeds or individual animals, may influence the type and emotional impact of interactions. As a result, the strength and nature of the associations between pet interaction and momentary affect observed in this study could vary depending on the characteristics of the companion animal, which were not captured in the present measures. Fifth, situations in which both a dog and a cat were present simultaneously were excluded from the analyses to ensure analytical clarity and to isolate species-specific effects without confounding influences of co-occurring interactions. Although this approach strengthens internal interpretability, it may reduce ecological validity and limit generalizability, particularly to multi-pet households where interactions with multiple animals are common. In such contexts, the emotional impact of human–animal interaction may differ from single-species encounters, potentially reflecting additive, buffering, or interaction effects. Finally, the last limitation relates to the way key constructs were measured using single items. Social support from companion animals was not measured directly; pet interaction was used as a proxy. Although prior evidence suggests that companion animals provide social support (McConnell et al., 2011; Beetz et al., 2012; Ståhl et al., 2023), the specific type and level of support in daily interactions remain unclear. In addition, human–animal interaction was assessed with a single EMA item. While this the use of single items is common in intensive longitudinal designs and helps reduce participant burden, it limits the ability to differentiate between interaction types, duration, or subjective meaning.

Future research should aim to refine and expand upon the current findings by addressing several methodological and conceptual limitations. For example, recruiting more balanced samples across pet types, breeds, and combinations of companion animals could clarify whether the observed patterns are robust across subgroups. Integrating physiological measures, such as heart rate variability, cortisol levels, or skin conductance, alongside EMA could help elucidate the mechanisms underlying the emotional responses observed, providing insight into how companion animals influence both affective and physiological stress reactions (Beetz et al., 2012). Differentiating between types of pet interactions, such as active play, training, or passive companionship, may uncover which aspects of interaction are most beneficial or potentially stressful, and whether these effects vary by context, time of day, or individual characteristics of the owner. Employing a time-lagged EMA design would allow researchers to examine temporal sequences and potential causal pathways between pet interactions and emotional states, providing insight into whether companion animals actively buffer stress or whether individuals in positive emotional states are more likely to engage with their companion animals. Finally, investigating species-specific mechanisms and multi-pet contexts would build on the nuanced findings in this study, such as the differential effect of cats versus dogs on negative affect, and clarify whether additive or interactive effects occur in real-world households settings. Together, these approaches would deepen understanding of the nuanced conditions under which companion animals influence human wellbeing.

## 5 Conclusion

This study demonstrates that interactions with companion animals are associated with momentary emotional wellbeing in daily life. Across species, interacting with a companion animal was linked to higher positive affect and lower negative affect, underscoring the emotional relevance of companion animals in everyday contexts. Importantly, these general emotional benefits were not species-specific, suggesting that both dogs and cats can support momentary wellbeing. In contrast, contrary to our expectations, the findings indicate that stress-buffering by interacting with a companion animal is not an underlying mechanism in this sample. Neither interacting with dogs nor cats buffered against the affective response to stressful events or activities. These findings align with the broader literature, which consistently reports heterogeneous and context-dependent findings regarding the effects of companion animals on wellbeing and stress.

This study adds to a growing body of EMA research suggesting that everyday interactions with companion animals are associated with momentary improvements in wellbeing. By capturing experiences in real time and in naturalistic contexts, the results support the idea that human–animal interactions can contribute positively to people’s daily emotional states. At the same time, the findings suggest that stress-buffering is unlikely to be the primary mechanism underlying the association between interaction with a companion animal and wellbeing in daily life. This does not mean that stress-buffering processes play no role in other forms of human–animal interaction. However, in the case of direct interaction with a companion animal, the results show that these interactions may not primarily moderate the impact of stress. Rather, they may more directly enhance positive affect and momentary wellbeing. Together, these findings highlight the value of examining human–animal interactions in everyday contexts and point to the need for further research into the mechanisms through which companion animals influence human wellbeing.

## Data Availability

The datasets presented in this article are not readily available because of privacy restrictions. Requests to access the datasets should be directed to mayke.janssens@ou.nl.
